# High Burden of Unrecognized Atrial Fibrillation in Rural India: An Innovative Community-Based Cross-Sectional Screening Program

**DOI:** 10.2196/publichealth.6517

**Published:** 2016-10-13

**Authors:** Apurv Soni, Allison Earon, Anna Handorf, Nisha Fahey, Kandarp Talati, John Bostrom, Ki Chon, Craig Napolitano, Michael Chin, John Sullivan, Shyamsundar Raithatha, Robert Goldberg, Somashekhar Nimbalkar, Jeroan Allison, Sunil Thanvi, David McManus

**Affiliations:** ^1^University of Massachusetts Medical SchoolWorcester, MAUnited States; ^2^Pramukhswami Medical CollegeKaramsadIndia; ^3^University of ConnecticutStorrs, CTUnited States

**Keywords:** atrial fibrillation, India, screening, mobile technology, community health workers

## Abstract

**Background:**

Atrial fibrillation, the world’s most common arrhythmia, is a leading risk factor for stroke, a disease striking nearly 1.6 million Indians annually. Early detection and management of atrial fibrillation is a promising opportunity to prevent stroke but widespread screening programs in limited resource settings using conventional methods is difficult and costly.

**Objective:**

The objective of this study is to screen people for atrial fibrillation in rural western India using a US Food and Drug Administration-approved single-lead electrocardiography device, Alivecor.

**Methods:**

Residents from 6 villages in Anand District, Gujarat, India, comprised the base population. After obtaining informed consent, a team of trained research coordinators and community health workers enrolled a total of 354 participants aged 50 years and older and screened them at their residences using Alivecor for 2 minutes on 5 consecutive days over a period of 6 weeks beginning June, 2015.

**Results:**

Almost two-thirds of study participants were 55 years or older, nearly half were female, one-third did not receive any formal education, and more than one-half were from households earning less than US $2 per day. Twelve participants screened positive for atrial fibrillation yielding a sample prevalence of 5.1% (95% CI 2.7-8.7). Only one participant had persistent atrial fibrillation throughout all of the screenings, and 9 screened positive only once.

**Conclusions:**

Our study suggests a prevalence of atrial fibrillation in this Indian region (5.1%) that is markedly higher than has been previously reported in India and similar to the prevalence estimates reported in studies of persons from North America and Europe. Historically low reported burden of atrial fibrillation among individuals from low and middle-income countries may be due to a lack of routine screening. Mobile technologies may help overcome resource limitations for atrial fibrillation screening in underserved and low-resource settings.

## Introduction

Atrial fibrillation is the world’s most common cardiac arrhythmia and, if untreated, increases the risk of stroke by upwards of five-fold [1]. Atrial fibrillation–related complications, particularly stroke, have reached epidemic proportions in low and middle-income countries. This is particularly true in India, where approximately 1.6 million persons suffer a stroke annually [2]. A growing number of people in India are affected by risk factors for atrial fibrillation, including hypertension and diabetes mellitus [3], and the contribution of atrial fibrillation to the ongoing stroke epidemic in India is unclear and understudied [4]. In India, where the majority of health care costs are out of pocket [5], routine evaluations using conventional electrocardiography (ECG) to diagnose atrial fibrillation are not standard of care. Therefore, an understanding of the atrial fibrillation epidemiology becomes dependent on systematic screening programs. Single-time, point-of-care screening programs face difficulties of their own because of the paroxysmal and minimally symptomatic nature of the majority of atrial fibrillation cases.

Here we report findings of a study to screen people for atrial fibrillation in rural western India using a US Food and Drug Administration (FDA)-approved single-lead ECG device, Alivecor, to overcome traditional constraints of dysrhythmia screening [6].

## Methods

Residents from 6 different villages in Anand District, Gujarat, India, comprised the base population. These 6 villages were randomly selected from a list of 30 villages where our community health workers are present. Trained research coordinators worked with the community health workers who were familiar with the layout of their respective villages and enrolled 60 participants from each village. Villages in India are typically organized by occupation-based colonies (fariyahs), and an equal number of participants were recruited from all fariyahs. The residents of every third house in each fariyah were approached for enrollment through the use of a systematic random sample. After obtaining informed consent, a team of trained research coordinators and community health workers enrolled a total of 355 participants aged 50 years and older to participate in the study.

**Figure 1:**
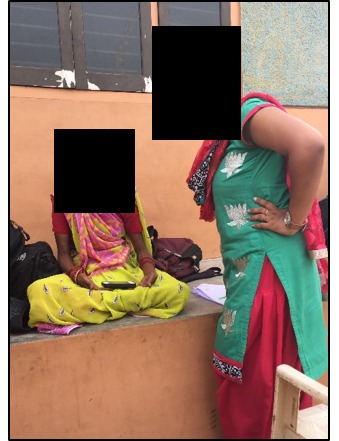
Community health worker screening a study participant for atrial fibrillation using a single-lead ECG device.

The study included two components: (1) screening using FDA-approved single-lead ECG device, Alivecor, and (2) collection of pulse data to develop an automated arrhythmia detection mobile app that can be used in low-resource settings [7,8]. Both Alivecor and pulse data were recorded serially for 2 minutes each on 5 consecutive days over a period of 6 weeks beginning June, 2015. During screening, participants sat cross-legged, resting the smartphone (iPhone 4S) in their lap to stabilize the phone and reduce excess motion that could interfere with the recordings (Figure 1). Additionally, on the day of enrollment, participants responded to a questionnaire that collected information related to their demographic characteristics, lifestyle habits, and past medical history.

**Figure 2:**
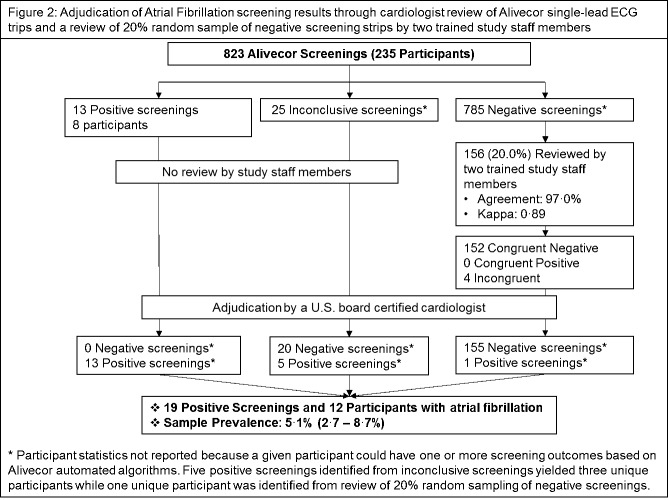
Adjudication of atrial fibrillation screening results.

The Alivecor device malfunctioned for two weeks, and therefore 120 participants from two villages were not screened for atrial fibrillation using Alivecor and were excluded from this study. Study staff uploaded ECG and pulse check recordings to a secure, Web-accessible Research Electronic Data Capture study database. Because pulse data were collected with the intention of developing an arrhythmia detection app based on the results of this pilot study, our outcome of atrial fibrillation was determined based solely on the ECG results from the FDA-approved Alivecor device. A board-certified cardiologist reviewed all ECG tracings for participants who had abnormal rhythm findings based on the automated Alivecor algorithm (Figure 2). Any participant found to have atrial fibrillation was referred to a study cardiologist located at a regional academic health center. Due to constraints in our available resources, our research staff did not follow up with participants after screening to assess whether any clinical plan was initiated.

A randomly selected 20% subsample of normal ECG tracings were reviewed by two trained study staff members, and discordant readings were adjudicated by the study cardiologist. Thus, a board-certified cardiologist reviewed the ECG tracings of all participants who were determined to have positive screening findings for the presence of atrial fibrillation. The study received institutional review board approval from the University of Massachusetts Medical School and HM Patel Center for Medical Care and Education. Descriptive statistics were utilized to describe the characteristics of study participants. Sociodemographic and comorbid factors were compared across different age groups using Fisher exact tests. Prevalence rates of atrial fibrillation were calculated in a standard manner with accompanying 95% confidence intervals. Given the limited sample size in our pilot investigation and the use of the Alivecor ECG as the source for gold standard measurement, we did not calculate performance measures.

## Results

Almost two-thirds of study participants were 55 years or older, nearly half were female, one-third did not receive any formal education, and more than one-half were from households earning less than US $2 per day (Table 1).

Twelve participants screened positive for atrial fibrillation yielding a sample prevalence of 5.1% (95% CI 2.7-8.7) (Figure 2); the characteristics of these individuals are shown in Table 2.

Only one participant had persistent atrial fibrillation throughout all of the screenings; 9 screened positive only once. The cumulative prevalence of atrial fibrillation in this population according to increasing number of screenings is presented in Table 3.

The first screening only identified 7 participants with a positive screen for atrial fibrillation. The remaining 5 participants who screened positive for atrial fibrillation were identified at the fourth screening. A comparison of the 235 participants included in the analyses, with the 120 excluded participants, revealed no meaningful differences between the two groups (see Multimedia Appendix 1 for details).

**Table 1:** Sociodemographic, lifestyle, and health characteristics of 234 participants from rural Gujarat, India, screened for arrhythmias, stratified by age groups.

			Age group (%)			
		N	50-55	55-65	65+	*P* value^a^
Female^b^		140	71.4	55.0	56.3	.09
**Education**						.19
	None	70	30.7	27.0	35.1	
	10^th^grade or less	129	50.0	59.6	58.4	
	More than 10^th^grade	29	19.4	13.5	6.5	
Works for pay		60	45.8	29.9	9.1	<.001
**Daily household income^c^**						.03
	Less than $1	71	33.9	28.1	31.7	
	$1-$2	62	14.5	31.5	31.7	
	$2-$4	53	37.1	20.2	15.2	
	More than $4	44	14.5	20.2	21.5	
Smoking history		37	7.9	14.3	11.3	0.25
Chew tobacco		58	33.3	22.0	21.3	0.03
Hypertension		87	27.0	34.1	48.8	0.02
Diabetes		20	9.5	5.5	11.3	0.37
Hypercholesterolemia		21	4.8	8.8	12.5	0.30

^a^Fisher exact test.

^b^One participant had completed the screening and thus was included in the analyses but did not respond to the questionnaire.

^c^Based on a conservative exchange rate of 1 USD = 60 INR for 2015 calendar year.

**Table 2:** Characteristics of 12 atrial fibrillation positive cases identified by a cardiologist review of single-lead ECG recording.

	Gender	Age	Index positive^a^	# positive^b^	Smoking	Hypertension
1	Female	50-55	3	1/3	No	No
2	Female	55-60	1	1/3	No	No
3	Female	60-65	1	5/5	No	No
4	Female	60-65	1	2/5	No	Yes
5	Female	75-80	1	1/4	No	Yes
6	Female	80-85	1	3/4	No	No
7	Male	50-55	3	1/3	Yes	Yes
8	Male	55-60	1	1/1	Yes	No
9	Male	60-65	1	1/1	No	Yes
10	Male	70-75	4	1/5	Yes	No
11	Male	75-80	3	1/3	No	Yes
12	Male	75-80	4	1/5	No	Yes

^a^Refers to the number of screening when atrial fibrillation was first recognized.

^b^Refers to the total number of positive screenings for a given participant.

**Table 3:** Cumulative prevalence of atrial fibrillation by number of screenings.

Screening number	Cumulative prevalence (95% CI)
1	3.0 (1.2-6.1)
2	3.0 (1.2-6.1)
3	4.3 (2.1-7.7)
4	5.1 (2.7-8.7)
5	5.1 (2.7-8.7)

## Discussion

### Principal Findings

Our study suggests a prevalence of atrial fibrillation in this Indian region (5.1%) that is markedly higher than has been previously reported in India and similar to the prevalence estimates reported in studies of persons from North America and Europe [1,9,10]. This finding is noteworthy and challenges conventional wisdom that individuals of European descent have higher rates of atrial fibrillation than individuals of Asian descent [1].

Current understanding of the global epidemiology of atrial fibrillation is dependent on robust surveillance systems and high quality community-based studies, but there remains a paucity of such investigations outside of North America and Europe, particularly in countries with less developed health systems [10]. A 2012 meta-analysis of community-based screening studies identified only one study from India [10]. That study was conducted in a tribal Himalayan village and found only one case of atrial fibrillation among 984 screened individuals, a prevalence rate of 0.1% [9]. However, 94% of participants in that study were less than 65 years old and thus not representative of the age profile of typical atrial fibrillation patients. A recently published opportunistic screening study of festival attendees reported a slightly higher but still low prevalence of atrial fibrillation (0.5%) among individuals 50 years of age or older [11]. Reasons for the discrepancies between our results and prior studies may include the shortcomings of opportunistic screening efforts involving younger individuals and the use of a single spot-check for atrial fibrillation. Our approach, in contrast to the two prior studies in India, utilized a randomized home-based serial screening of participants aged 50 years and older in order to detect paroxysmal and persistent atrial fibrillation. The higher yield from multiple rhythm checks versus a single check for the detection of paroxysmal atrial fibrillation in the community has been emphasized by other studies [12] and is made evident by our findings. Namely, we observed that out of the 12 participants who screened positive for atrial fibrillation, only one had persistent atrial fibrillation. Moreover, 5 participants who were ultimately found to have paroxysmal atrial fibrillation did not have atrial fibrillation detected during their first screen.

Recently, there has been increased attention in North America and Europe to leverage mobile technology for the screening of persons with undetected atrial fibrillation [12,13]. The establishment of the National Programme for Prevention and Control of Stroke by the Indian government supports the importance of stroke prevention in India. However, due to the cost of ECG-based screening programs and paucity of trained health professionals in many regions, atrial fibrillation screening has not been possible to date. Our efforts suggest that by engaging community health workers to use novel mobile technologies for arrhythmia monitoring we can screen large numbers of Indians for atrial fibrillation. Our capacity to recruit and serially screen residents of the rural Anand community was strengthened by a long-standing relationship between investigators and community health workers in India.

### Limitations

The findings of our study need to be interpreted with appropriate caution given several concerns and limitations. First, this study is based on a relatively small sample size of 235 participants. Therefore, we have presented information about sample sizes and accompanying 95% confidence intervals to demonstrate the range of possible prevalence estimates consistent with the variability observed in our data. Second, we did not perform a gold standard 12-lead ECG to confirm our positive screening findings. It is important to note, however, that Alivecor devices are FDA-approved and are widely used by cardiologists in diverse clinical settings [14]. Lastly, our cross-sectional study design limits our ability to assess any potential outcomes associated with atrial fibrillation or characterize the clinical presentation of atrial fibrillation in more detail. Therefore, future efforts should explore the feasibility and costs associated with replicating our approach in other environments to define the accuracy of the automated algorithms employed in larger and more diverse cohorts, to create referral mechanisms which can accommodate newly identified patients, to more systematically characterize the clinical presentation of atrial fibrillation (eg, valvular diseases, comorbidities, psychosocial impact), and to demonstrate reduced stroke rates through the primary prevention of stroke in screened populations.

### Conclusions

In conclusion, our study has two important implications: (1) mobile technologies may help overcome resource limitations for screening adults for atrial fibrillation in underserved and low-resource settings and (2) serial screening for atrial fibrillation enhances the ability to identify persons at risk for atrial fibrillation.

## Multimedia Appendix 1

Comparison of sociodemographic, lifestyle, and health characteristics of participants that were excluded versus included in the study.

## Abbreviations

ECG: electrocardiogram
FDA: US Food and Drug Administration
