# Supplementary material for: High Burden of Unrecognized Atrial Fibrillation in Rural India: An Innovative Community-Based Cross-Sectional Screening Program
Source: JMIR Public Health Surveill. 2016 Oct 13;2(2):e159. doi: 10.2196/publichealth.6517 (PMC5083844; doi:10.2196/publichealth.6517)
Supplement: Supplementary file 1 [file publichealth_v2i2e159_app1.pdf]

Supplemental Table 1: Comparison of sociodemographic, lifestyle, and health characteristics of participants that were excluded vs. included in the study

	Excluded	Included	p
**Number**	119[a]	234[b]	
**Age**			
50-55	27.7	26.9	
55-65	43.7	38.9	0.54
65+	28.6	34.2	
**Female**	69.7	60.0	0.09
**Education**			
None	43.9	31.0	
10th Grade or less	49.1	56.3	0.04
More than 10th Grade	7.0	12.7	
**Works for pay**	23.2	26.8	0.50
**Daily household income[b]**			
Less than $1	37.3	30.7	
$1-2	19.5	27.3	0.32
$2-4	26.3	22.9	
>$4	16.9	19.1	
**Smoking history**	7.6	11.5	0.51
**Chew tobacco**	30.3	23.4	0.35
**Hypertension**	31.1	37.0	0.27
**Diabetes**	12.6	8.5	0.22
**Hypercholesterolemia**	10.1	8.9	0.73

a: one participant that was excluded from the final analyses had not responded to the questionnaire
b: one participant had completed the screening and thus was included in the analyses but did not respond to the questionnaire
